# Introducing cell-free DNA noninvasive testing in a Down syndrome public health screening program: a budget impact analysis

**DOI:** 10.1186/s12962-020-00245-5

**Published:** 2020-11-04

**Authors:** L. Nshimyumukiza, J. A. Beaumont, F. Rousseau, D. Reinharz

**Affiliations:** 1 Département de médecine sociale et préventive, Faculté de Médecine, Université Laval, Pavillon Ferdinand Vandry, Local 2432, 1050 Avenue de La Médecine, Quebec, QC G7V0A6 Canada; 2 Département d’informatique et de Génie Logiciel, Faculté de Sciences et de Génie, Université Laval, Quebec, QC Canada; 3 Centre de Recherche du Centre Hospitalier Universitaire de Québec, Québec, QC Canada; 4 Département de Biologie Moléculaire, Biochimie Médicale et Pathologie, Faculté de Médecine, Université Laval, Quebec, QC Canada

**Keywords:** Budget impact analysis, Cell-free DNA, Non-invasive prenatal screening, Trisomy 21

## Abstract

**Background:**

Non-invasive prenatal testing (NIPT) using cell-free fetal DNA in maternal plasma is a high accurate test for prenatal screening for Down syndrome. Although it has been reported to be cost effective as a contingent test, evidence about its budget impact is lacking.

**Objective:**

To evaluate, using computer simulations, the budget impact of implementing NIPT as a contingent test in the Quebec Program of screening for Trisomy 21.

**Methods:**

A semi-Markov analytic model built to simulate the budget impact of implementing NIPT into the current Quebec Trisomy 21 public Prenatal Screening, Serum Integrated prenatal screening (SIPS). Comparisons were made for a virtual population similar to that of expected Quebec pregnant women in 2015 in terms of size and age. Data input parameters were retrieved from a thorough literature search and in government databases, especially data from Quebec Program of screening for Trisomy 21. The 2015–2016 fiscal year budget impact was estimated from the Quebec healthcare system perspective and was expressed as the difference in the overall costs between the two alternatives (SIPS minus SPS + NIPT).

**Results:**

Our study found that, at a baseline cost for NIPT of CAD$ 795, NIPT as a second-tier test offered to high-risk women identified by current screening program (SIPS + NIPT) may be affordable for Quebec health care system. Compared to the current screening program, it would be implemented at a neutral cost, considering a modest annual savings of $ 80,432 (95% CI $ 79, $ 874–$ 81,462). Results were sensitive to the NIPT costs and the uptake-rate of invasive diagnostic tests.

**Conclusion:**

Introducing NIPT as a contingent test in the Quebec Trisomy 21 screening program is an affordable strategy compared to the current practice. Further research is needed to confirm if our results can be reproduced in other healthcare jurisdictions.

## Introduction

Noninvasive prenatal testing (NIPT) using cell-free fetal DNA (cffDNA) circulating in maternal blood is a highly accurate screening test for Down syndrome (DS) and two other common aneuploidies, namely Trisomy 18 (T18) and Trisomy 13 (T13), both in high risk and in general populations of pregnant women [1, 2]. Its detection rates (DR) are around 99% for DS, 97% for T18 and T13 whereas the false positive rate (FPR) is about 0.1% for these aneuploidies [1, 2]. Therefore, NIPT provides an opportunity to improve aneuploidy detection and to reduce the use of invasive diagnostic procedures as well as their related miscarriages. Several studies have effectively suggested that the introduction of NIPT has led to a significant reduction of invasive diagnostic procedures compared to current screening practice [3–6].

In Quebec, the Quebec Ministry of Health and Social Services published the framework of a fully reimbursed Prenatal Screening Program for Trisomy 21 in 2011. The objective of this program is to provide to all pregnant women, regardless of age, a prenatal screening for Trisomy 21 within the public health insurance scheme [7]. Quebec’s Health Insurance Plan is a public plan that gives all Quebecers access to free health care by covering a wide range of medical care, including almost all medically necessary care provided by a family doctor, a general practitioner or a specialist. The plan is managed by *the Régie de l’assurance maladie du Québec* (health insurance board), also known as the “RAMQ”.

The screening program offers the serum integrated screening approach (SIPS) which consists of the integration of biological measurements performed at two periods of a pregnancy: first trimester pregnancy-associated plasma protein A (PAPP-A) and second trimester quad markers in two separate blood tests. Quad markers include alpha-fetoprotein (AFP), unconjugated estriol (uE3), human chorionic gonadotropin (hCG) and inhibin-A. The first blood test is collected between 9 and 13 + 6 weeks (best at 10–11 + 6 weeks) and the second between 14 and 20 + 6 weeks (best at 15–16 weeks). Then, with combination of maternal age, a single result is computed with a trisomy 21 risk cut-off of 1 in 3007 and amniocentesis as confirmatory invasive test is scheduled at 16 weeks for those with a positive screen (risk ≥ 1/300). The program recommends also, when available, the integration of nuchal translucency measurements results in the risk calculation. The Quebec screening program for trisomy 21 is only indicated for woman with singleton pregnancy. The prenatal screening and follow-up for woman with multiple pregnancy is left to the judgement of doctors. In addition, according to the recommendations of the Society of Obstetricians and Gynecologists of Canada (SOGC) [8], the Quebec program does not recommend an invasive prenatal diagnosis (e.g. amniocentesis) based solely on age in the absence of screening results.

A recent evaluation of this program found that about 4.5% of pregnant women who participated in the screening program have positive results, the majority being false positives. This high rate of false positive results leads to a high number of unnecessary amniocenteses [9]. Having highly accurate non-invasive prenatal screening test such as NIPT is therefore of interest for the program. Nevertheless, in the context of limited resources, decision-makers need information on health economic aspects (cost-effectiveness and budget impact analysis) of NIPT before considering its implementation into a universal prenatal screening program.

Among the heterogeneity of cost-effectiveness results on NIPT compared to current screening practice, there are several studies that suggest that the NIPT as a contingent test is likely to be cost effective [10–20]. Besides, the society of obstetricians and gynaecologists of Canada recommends the use of NIPT for women at high risk of Down’s syndrome before proceeding to an invasive confirmation tests [21]. Yet, a budget impact analysis is still needed. This necessary information in support of the decisions to make regarding the relevance and sustainability of introduction NIPT as a contingent test in the Quebec prenatal screening for trisomy 21 and its reimbursement by the public health system is still lacking.

## Methodology

### Model overview

A semi-Markov agent-based analytic model using the Clumeq supercomputer network-running SCHNAPS platform simulator [22–24] was built to simulate the budget impact of implementing NIPT into the current Quebec Prenatal Screening program for trisomy 21. A virtual cohort of pregnant women similar to that of Quebec in 2015 in terms of age and pregnancy rate by age was constructed. Each fetus was assigned a Down syndrome status (yes or no) based on maternal-age-specific risk in the first trimester of pregnancy [25, 26] at the beginning of prenatal screening (12 weeks of pregnancy). Our microsimulation allows each woman to be followed in the model on a weekly cycle’s basis during pregnancy up to birth in order to well take into account the risks of spontaneous losses and voluntary induced abortion. Indeed, clinical evidence have shown that the risk of spontaneous pregnancy loss is higher in the first trimester than in the second and in third trimester [27–31]. In addition, in Quebec, the proportion of voluntary induced abortion are high ranging between 20 and 25% of all pregnancies.

The 2015–2016 fiscal year budget impact was estimated from the Quebec healthcare system perspective. As suggested by the budget impact analysis principles of good practice [24], only undiscounted directs costs were considered. The budget impact was expressed as the difference between the two alternatives (SIPS minus SIPS + NIPT). Although costs could be disaggregated into various components (screening, invasive diagnostic tests and other costs) it is the overall cost that was considered as the principal outcome.

### Model decision structure

**Fig. 1:**
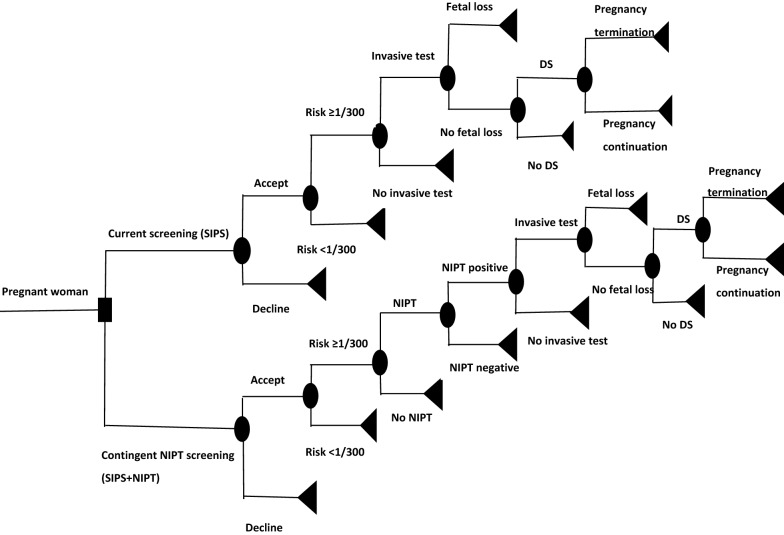
Simplified decision model of current screening practice and Contingent NIPT (NIPT following a risk positive current screening practice)

The simplified decision model is depicted in Fig. 1. To simplify the model, we made some assumptions:

- The model considers only prenatal screening for singleton pregnancies as recommended in Quebec.
- Pregnant women who decline prenatal screening follow the natural course of pregnancy.
- As the DS risk calculation is scheduled in the second trimester of pregnancy (after QUAD test), amniocentesis was considered as the only invasive test used by the Quebec prenatal screening for DS. Its sensitivity and specificity were considered as 100%.
- Pregnant women who decline diagnostic testing (amniocentesis) after positive results from current prenatal screening or contingent NIPT follow the natural course of pregnancy.
- As we assumed that cost of pregnancy follow-up does not differ between screening strategies compared, only costs related to screening and diagnosis as well as their short-term consequences were considered.
- The pregnant women would comply with the scheduled second trimester QUAD test unless their pregnancy was terminated (voluntary induced abortion or spontaneous loss).
- Genetic counselling is offered to all pregnant women with confirmed DS after screening and diagnostic.
- All pregnant women with a failure NIPT test result are retested.

Two screening pathways, namely current practice (SIPS) and Contingent NIPT (SIPS + NIPT) are compared. In the “Current practice” pathway, a pregnant woman has a probability of accepting or declining the screening offer. For women who accept, a screening by SIPS is positive if DS risk is ≥ 1/300. Then, the model assigns to the pregnant woman a probability to undertake a diagnostic invasive test (amniocentesis) to which is associated a risk of procedure-related fetal loss or amniotic fluid leakage. If the result of the invasive test is positive, the woman can decide to terminate or not her pregnancy.

In the “Contingent NIPT” pathway, a pregnant woman has also a probability of accepting or declining the screening offer. For women who accept, the SIPS is offered. If the risk is ≥ 1/300, NIPT test is proposed. The pregnant woman has a probability to accept or to decline NIPT. For those who accept NIPT, if the test result is positive, the model assigns to the pregnant woman a probability to undertake a diagnostic invasive test (amniocentesis) to which is associated a risk of procedure-related fetal loss or amniotic fluid leakage. If the result of the invasive test is positive, the woman can decide to terminate or not her pregnancy. The model assumes that if the result of NIPT test is negative, there is no further testing. In addition The model takes into account the probability of NIPT failure [32] and thus the possibility of NIPT retest.

### Input parameters

#### Population and events probabilities

Population and events probabilities data are presented in Table 1. A virtual population of 15–49 years old singleton pregnant women was generated and calibrated using Quebec statistics data [33, 34]. This population is followed weekly by taking into account the risks of spontaneous losses and voluntary pregnancy termination [27–31] as well as the age-specific risk of DS [25, 26]. Input data used are based on the Prenatal Screening Program of Quebec for trisomy 21 data [9] and on published sources. Following events were considered: consent to participate in aneuploidy prenatal screening program [9]; performance of screening tests [1, 2, 35–37]; consent to NIPT after a positive screening result with current approaches [38]; NIPT failure rates [32]; consent to undergo invasive testing [9] procedure-related loss (risk of miscarriage after amniocentesis) [39]; amniotic fluid leakage [40] and termination of pregnancy when DS is confirmed [41, 42].

**Table 1:** Input model parameters

Parameter	Baseline	Values for sensitivity analysis	Source
Population			
Number of singleton pregnant women 15–49 years	111,752	111,003–113,134	Generated and calibrated using [1, 2]
Down syndrome risk by maternal age	0.10 to 6.6%		[3]
Voluntary induced abortion risk in Quebec	23% (90% in first trimester and 10% in second trimester)		[4]
Spontaneous fetal loss risk, unaffected pregnancies	13.5%		[5]
Spontaneous fetal loss risk, DS affected pregnancies	Week 10: 36%; week 12: 30%; week 14: 25%; week 16: 21%		[3, 6]
Uptake rates			
Current practice	50%	50–90%	[7]
NIPT	90%	70–95%	[8, 9]
Amniocentesis	90%	70–95%	[10, 11]
Termination of pregnancy, DS confirmed	90%	50–100%	[10, 11]
Screening performances and outcomes			
Current screening practice			
Detection rate	85%	75–90%	[12]
False positive	3.3%	2.5–5%	[7]
NIPT			
Detection rate	99.9%		[13, 14]
False positive rate	0.1%		
Failure rate of NIPT testing	2%	0–4%	[15]
Fetal loss from amniocentesis	0.11%	0.05–1%	[16]
Amniotic fluid leakage	1%	0.5–2%	[17]

#### Costs

Costs were estimated in Canadian dollars for the fiscal year 2015–2016. They are presented in Table 2. All costs reflect the direct healthcare costs from the perspective of the Quebec public healthcare system. Cost components included costs of services associated with DS screening (genetic counseling, screening and invasive tests) and of medical services related to the following events: procedure-related loss (risk of miscarriage after amniocentesis); rupture of membranes with amniotic fluid leakage; termination of pregnancy in case of DS. One-time costs related to the implementation of NIPT were not considered for two reasons: (1) we considered a price for commercial kits which already take into account the investment incurred; (2) its introduction as a contingent test allows it to easily integrate and use the resources (human and material) of the existing prenatal screening program.

**Table 2:** Unit costs (CAD$)

Items	Baseline unit cost (CAD$)	Values for sensitivity analyses	Source
Current screening practice	108.60		[14, 42, 46]
NIPT (including interpretation)	795	100–1000	[19, 48]
Amniocentesis (including karyotype and its interpretation)	864.39	600–1000	[14, 42, 47]
Genetic counselling	138.636		[14]
Program administration	18.07		[14, 45, 49]
Termination of pregnancy in case of confirmed DS	1,632,48		
Amniocentesis related loss	291,900		
Hospitalization after amniotic fluid leakage related to amniocentesis	297,100		

All unit prices were Quebec public provincial average prices calculated from governmental databases. Unit prices for clinical activity centers were calculated using the 2015–2016 Annual financial reports of four hospitals involved in T21 screening in Quebec (*CHU de Québec, CHU de Sherbrooke, Centre universitaire de santé McGill and CHU Sainte Justine*) [43]. In the Quebec healthcare system, unit prices are based on the *NIRRU*–*Niveau d’intensité relative des ressources utilisées* (relative intensity level of resources used that is attributed to each type of medical procedure but exclude medical fees. An overhead premium was added to all unit prices in order to capture the costs of the ancillary activity centers (e.g. laundry services, archives, etc.) which support all clinical units in the hospital. The overhead was calculated using direct method i.e. the share of ancillary services allocated to a clinical activity center is proportional to the weight of this clinical center in the hospital [44]. For NIPT, we considered a price of CAD$ 795 per test [14, 20] in the base case analyses but a wide range of prices were used in sensitivity analyses. The lowest price considered for this test was 100 CAD$. Medical fees related to medical interventions and genetic counselling were the cost paid by the provincial public health insurance, the *Régie d’assurance maladie du Québec* (RAMQ) [45]. The “All Patient Refined Diagnosis Related Groups” (APR-DRG) database was used to calculate the average price of the hospital resources consumed by a pregnant woman who required a hospitalization [46].

### Sensitivity analyses

As suggested by the principles of good practice for budget impact analysis [24], we performed the scenario analyses by changing selected key input parameters in order to produce plausible alternative scenarios. The univariate sensitivity analyses were performed with parameters that were considered to possibly influence the difference of costs between the Contingent NIPT option and the current screening practice. These parameters are: current practice screening uptake (50–90%), probability of positive screen following current screening practice (2.6–5%), invasive testing uptake (70–95%), NIPT uptake (70–95%), invasive testing price ($ 600–$ 1000), and NIPT price ($ 100–$ 1000). The model also tested the scenario of offering the choice between contingent NIPT and the direct invasive test after a positive screen (risk ≥ 1/300), a scenario observed in UK where 20% of pregnant women with positive screen prefer undergoing directly to invasive test [38].

### Simulation and validation

The model was validated and calibrated using Bayesian methods by Markov Chain Monte Carlo (MCMC) simulations [47, 48]. Simulations for each option were repeated 1000 times, each time on a newly generated (i.e., different) independent virtual population. Validation was performed by comparing the results with expected published or assumed values. As example, our model predicted 111,752 pregnancies (95% CI 111,003–113,134) corresponding to a pregnancy rate of 5.95% (95% CI 5.9–6.1%) which is very close to what was expected (i.e. pregnancy rate of 6%) closer to that observed in last years [9].

## Results

In the base case scenarios where NIPT test cost is set at CAD$ 795 and first screening step (SIPS) uptake is 50% as well as a NIPT uptake of 90%, the NIPT as second tier option (Contingent NIPT) comes out as a cost neutral or cost-saving screening strategy compared to the current screening approach (SIPS) for DS. Indeed, results suggest that in population of singleton pregnant women of 111,752 individuals (95% CI 111,003–113 134), replacing the current DS screening strategy by the contingent NIPT screening strategy would allow a minor saving of 1.6% or $ 80,432 (95% CI $ 79,874–$ 81,462) (Table 3).

**Table 3:** Budget impact analysis of Contingent NIPT(SIPS + NIPT) compared to current screening practice (SIPS): baseline results

Items	Number of procedures		Costs (CAD$)		Costs difference (Contingent NIPT minus Current practice)
	Current practice (SIPS)	Contingent NIPT (SIPS + NIPT)	Current practice (SIPS)	Contingent NIPT (SIPS + NIPT)	
Screening tests					
SIPS	41,904	41,904	4,550,887	4,550,887	$ 0
NIPT	0.00	1809	0	1,437,863	$ +1,437,863
Total for screening tests			4,550,887	5,988,750	$ +1,437,863
Invasive tests (Amniocentesis and karyotype)	1773	103	1,532,710	89,420	$ −1,443,290
Other procedures					
Genetic counselling	114	101	15,777	14,049	$ −1728
Procedure related loss	2	0.1	5693	332	$ −5361
Hospitalization for amniotic fluid leakage	18	1	52,681	3073	$ −49,607
Termination of pregnancy (Confirmed DS)	102	91	167,199	148,890	$ −18,309
Program administration	41,904	41,904	757,339	757,339	$ 0
Total for other procedures			998,688	923,683	$ −75,005
Overall			7,082,285	7,001,853	$ −80,432 95% CI ($ −81,462 to $ −79,874)

**Fig. 2:**
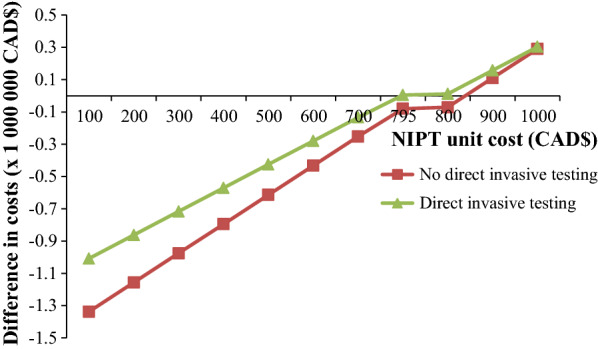
Marginal costs between contingent NIPT (with or without direct invasive testing) vs current practice by varying NIPT unit cost

The results of the sensitivity analyses show that the Contingent NIPT screening strategy remains cost-saving or cost-neutral except when the cost and the uptake of invasive testing (amniocentesis) are low or when the cost of NIPT is high (Table 4). The two strategies have a quite similar cost under conditions where 72.5% of women with current practice positive screen choose NIPT and 20% choose direct invasive testing (Fig. 2). When the cost of NIPT is further lowered, the net savings from Contingent NIPT become substantial (up to 20% or 1.4 million CAD$), especially if the possibility of choosing direct invasive testing i.e. without a prior NIPT) was not offered (Fig. 2).

**Table 4:** Marginal costs between contingent NIPT vs current practice by varying key input parameters: univariate sensitivity analyses results

Parameter	Value	Total costs current practice (CAD$)	Total costs Contingent NIPT (CAD$)	Costs difference (Contingent NIPT minus Current practice) ($)
Cost NIPT	1000	7,082,285	7,372,622	290,337
	100	7,082,285	5,744,853	−1,337,432
Cost amniocentesis	1000	7,322,744	7,015,881	−306,863
	600	6,613,477	6,974,502	361,025
Positive screen SIPS	5%	7,360,645	7,007,644	−353,00
	2,50%	6,455,110	6,987,523	532,413
Amniocentesis uptake	95%	7,167,436	7,003,767	−163,669
	60%	6,571,382	6,993,807	422,425
NIPT uptake	95%	7,082,285	7,086,891	4606
	60%	7,082,285	6,491,623	−59,066
Screening participation uptake	90%	8,960,783	8,832,034	−128,749
	40%	6,612,494	6,544,141	−68,353

## Discussion

The objective of the present study was to evaluate the budgetary impact of introducing NIPT as a contingent test in the Quebec Trisomy 21 screening program. We evaluated the budget impact to support of the decisions to make regarding the relevance and sustainability of introduction NIPT as a contingent test in the Quebec prenatal screening for trisomy 21. Indeed, some studies have reported that cost-effectiveness projects can have little influence on the healthcare decision-making process [49]. This is explained by the fact that economic evaluations, especially cost-effectiveness and cost-utility analyses, tend to focus on the relative efficiency of the new intervention compared to its comparator and not on budget impact. Budget impact analyses have emerged as a useful tool to answer this problem, as they focus on the question of affordability of the new intervention compared to the cost of the comparator. They therefore provide valuable additional information to decision-makers who are budget holders [50].

To our knowledge, this is one of the few to report a budget impact analysis of the introduction of NIPT as a contingent test into a national DS screening program in Canada. Previous studies mainly consisted of cost-effectiveness studies [10–20, 51].

Our results show that the Contingent NIPT is a cost-saving or a cost-neutral option. This result is explained by the fact that the additional costs due to NIPT are compensated by the savings due to reduction of the number of invasive tests (see Table 4). Reducing further the cost of NIPT obviously increases the savings in the screening strategy involving NIPT (Fig. 2). However, when the cost of the invasive test is lower than the cost of NIPT, the contingent NIPT option becomes more costly than the current practice.

Considering that previous studies performed on NIPT have not focused on budget impacts but on the cost-effectiveness, the comparison with other studies is limited. However, our results provide an additional argument in favor of introducing NIPT as a contingent test in in national DS screening programs.

This study has some limitations. First, although decision analytical model and simulations are useful tools that can contribute to clarifying the expected costs and benefits of the interventions, a certain simplification of the reality is needed, and assumptions had to be made on the model parameters. For this, data from the Quebec heath care system of from the review of the literature were used but for some. E.g. assumptions had to be made regarding the uptake rate of NIPT as this technology is not yet implemented into universal screening programs. Data used had to be taken from proxies. We cannot guarantee that in the real life, the same percentage would have been observed. However, we believe that the extensive sensitivity analyses done in this study allowed us to handle this issue.

The second limitation is related to the costs included in our analyses. Indeed, the one-time costs for investment and the implementation of NIPT were excluded and only direct costs related to the screening and diagnosis as well as their short-time consequences were included on basis of the duration time of pregnancy. The total costs of the Contingent NIPT option might therefore be higher than of the cost of the current practice option in the first years of its implementation. However, as stated in “Methodology” section, in our analysis, we considered a high cost equivalent to that of commercial kits which already consider the investment incurred. Also, as its introduction as a contingent test allows it to be easily integrated and use the resources (human and material) of the existing prenatal screening program, we believe that this extra cost could be compensated by resources devoted to cytologist services that could be released as the need for prenatal cytogenetics will continue to fall with the reduction of invasive procedures.

Despite these limitations, this study suggests that introducing NIPT as a contingent test in the Quebec Trisomy 21 screening program is an affordable strategy compared to the current practice. Beside this affordability, one should note that it can be easily implemented in clinical practice as an add-on to the current Quebec practice (PAPP-A in the first trimester and hCG, AFP, uE3 and inhibin A in second trimester screening) for better selecting pregnant women who should undergo invasive test.

Finally, one should stress the fact that our results apply to the Quebec healthcare system (a quasi-exclusive public healthcare system). They might not be generalizable to other settings. Further research is needed to confirm if our results can be reproduced in other healthcare jurisdictions. However, some of our sensitivity analyses provide estimates of how NIPT could impact other jurisdictions since pregnancy trajectories are similar.

## Data Availability

Authors confirm that all relevant data are included in the article.
